# Prediction scenarios of past, present, and future environmental suitability for the Mediterranean species *Arbutus unedo* L.

**DOI:** 10.1038/s41598-021-03996-0

**Published:** 2022-01-07

**Authors:** Alice Maria Almeida, Maria João Martins, Manuel Lameiras Campagnolo, Paulo Fernandez, Teresa Albuquerque, Saki Gerassis, José Carlos Gonçalves, Maria Margarida Ribeiro

**Affiliations:** 1 Instituto Politécnico de Castelo Branco, Castelo Branco, Portugal; 2 C4 - Centro de Competências Em Cloud Computing (C4-UBI), Universidade da Beira Interior, Covilhã, Portugal; 3 Forest Research Centre, Instituto Superior de Agronomia, Tapada da Ajuda, Lisbon, Portugal; 4 Departamento de Ciências e Engenharia de Biossistemas, Instituto Superior de Agronomia, Tapada da Ajuda, Lisbon, Portugal; 5 MED – Mediterranean Institute for Agriculture, Environment and Development, Universidade de Évora, Évora, Portugal; 6 Centro de Recursos Naturais, Ambiente e Sociedade (CERNAS) - Instituto Politécnico de Castelo Branco, Castelo Branco, Portugal; 7 Instituto Ciências da Terra, Universidade de Évora, Largo dos Colegiais, Évora, Portugal; 8 Department of Natural Resources and Environmental Engineering, Universidade de Vigo, Lagoas, Marcosende, Vigo, Spain; 9 Centro de Biotecnologia de Plantas da Beira Interior, Quinta da Senhora de Mércules, Castelo Branco, Portugal

**Keywords:** Ecology, Climate sciences, Ecology

## Abstract

Climate change is a challenge for forests in the coming decades, with a major impact on species adaptation and distribution. The Mediterranean Basin is one of the most vulnerable hotspots for biodiversity conservation under climate change in the world. This research aimed at studying a Mediterranean species well adapted to the region: the *Arbutus unedo* L. (strawberry tree). The MaxEnt, a presence-only species-distribution software, was used to model *A. unedo’s* environmental suitability. The current species potential distribution was accessed based on actual occurrences and selected environmental variables and subsequently projected for the Last Glacial Maximum (LGM), the Mid-Holocene (MH), and the years 2050 and 2070, considering the two Representative Concentration Pathways: RCP4.5 and RCP8.5. Results from the LGM projection suggest the presence of refugia in the core of the Mediterranean Basin, in particular the Iberian Peninsula (IP). The projections for the MH indicate increasing climatic suitability for the species and an eastward expansion, relatively to LGM. The predicted future environmental changes will most likely act as a catalyst for suitable habitat loss and a range shift towards the North is likely to occur.

## Introduction

Climate change will provoke increasing temperatures and drought, which allied with floods and forest fires will pose large challenges for the Mediterranean forest in the future, with direct consequences in the species’ presence and distribution. According to recently published studies^1,2^, climate models clearly show that the Mediterranean Basin is a climate change hotspot in the current century. Moreover, those authors predict that precipitation will decrease inversely with temperature, at a rate around 20 mm/K, and temperature will suffer a 20% increase, compared to the world global average in the last decade. The warming will be drastic in the summer (ca. 50% greater than global warming), especially for the land areas north of the Mediterranean Sea (up to 100% greater than global warming)^1^. In the future, climate change may lead to a change in the species’ geographical distribution, conducting to geographical ranges shifting^3^. Indeed, in a less suitable climate, tree species might respond by short-term acclimation, long-term adaptation, and altitudinal and latitudinal migrations^4^, due to habitat loss and shifts in the spatial distribution of climatic conditions^2^. Predictable suitability reduction in the Mediterranean landscape and consequent expansion of arid areas in a fragmented landscape may exacerbate the climate change effect on the Mediterranean region species^2^, and projected future precipitation changes, together with increasing temperatures, will have substantial effects on regional drought and fire events’ intensity/frequency^5^. Indeed, summer drought intensification in this region could be critical for certain species, particularly those in the southern range^6^. Therefore, forests can be negatively affected by the increase in drought intensity, frequency, or duration^7^.

Few large-scale studies were made so far with typical Mediterranean tree species, at their range level, in future global change scenarios^8,9^. The herein survey aims to contribute to a broader discussion about the impact of global change in a typical Mediterranean tree species, introducing the preliminary results obtained for *Arbutus unedo* L. (strawberry tree). The study of widely distributed species under broad climatic change scenarios is relevant to understand the environmental/habitat requirements, and effects on their development. The Mediterranean Basin is one of the earth's 25 biodiversity hotspots, characterized by a complex geological and palaeoclimatic history^2,10^. The climatic conditions in the Mediterranean Basin, with wet cold winters and hot dry summers, had a deep effect on plant species evolution. Thus, the climate change threat is a serious concern in biodiversity conservation in this region, which associated with the long-lasting anthropogenic influence, led to habitat fragmentation and destruction due to land-use pressure^2,11^.

Species distribution models (SDMs) aim to find associations between the species’ observed occurrences and the selected environmental variables. Thus allowing to characterize ecological niches and to map the potentially occupied areas. They are used to predict the species’ distribution, by adjusting relationships between environmental variables and known species incidence records to identify the environmental conditions necessary for the population viability, and may require extrapolation in space and time^12^. Those models are widely used to investigate both climate change effects on potential taxon spatial distribution and habitats’ environmental suitability, as well as understanding species’ potential environmental suitability in past climates^9,13–16^.

The core objective of this survey was to study the climate influence, in different time slices, on the spatial distribution of the species *Arbutus unedo*, a predominantly Mediterranean evergreen small tree from the Ericaceae family, an instance of sclerophyllous and dry-adapted flora^17,18^. The species’ distribution is largely within the Mediterranean Basin, according to the bioclimatic criteria, mainly observed in mild climates, coastal and inland areas, where summer dryness and winter frost were not too extreme in the past^19,20^. More specifically, the *A. unedo* has a mainly Mediterranean distribution, occupying a coastal strip from Tunisia to Morocco along the North of Africa, and from the Iberian Peninsula (IP) to Turkey, through southern Europe, northern IP, western France, and south-western Ireland^10,19,21,22^.

The *Arbutus unedo* occurs at altitudes between sea level and 1200 m, mainly on rocky slopes, unsuitable areas for woodland, with vegetation dominated by shrub communities, where it can compete and develop^23,24^. This species is unable to reproduce and compete when shaded, therefore it is frequent at the woodland edge, where, usually, has a bushy format^21^. Otherwise, *A. unedo* is ecologically versatile, growing on wide soil conditions, although with a siliceous or decarbonated substrata preference^19,21,22^. Additionally, the *A. unedo* plants are fire or graze resistant, since they can easily resprout^25^. Nevertheless, *A. unedo* was found to be more drought sensitive than other Mediterranean species (e.g. *Quercus ilex* L., *Phillyrea latifolia* L.) in a 15-year experimental-drought study^26^.

In this study, the MaxEnt software was used to compute and fit the best species’ distribution model aiming to evaluate the *A. unedo* potential suitability distribution in past, current, and future scenarios^27–29^. The specific objectives were to (1) identify and select the environmental variables that influence  the species distribution and MaxEnt model performance, (2) evaluate the current potential distribution range, and (3) project and predict potential distributions’ changes, according to the following climate scenarios: LGM, MH, and predictions for 2050 and 2070 under two Representative Concentration Pathways, the RCP4.5, and the RCP8.5^30^.The obtained results target to contribute to a better understanding of the biology/ecology of the species providing information for future management and conservation practices acting as an alert for the substantial impact of global climatic change in currently well-adapted species.

## Results

### Model evaluation and variable contribution

The selected topographic and environmental variables, resulting from a two-step procedure, are listed in Table 1. Firstly, the MaxEnt model was fitted using the 12 least correlated variables. Secondly, for each variable, the MaxEnt computed the respective percent contribution, the permutation importance, and, finally, the Jackknife test results. Afterwards, the 12 variables were ranked according to those criteria. Table 1 shows the values and ranks for the eight highest rated variables. Those chosen eight variables retained more than 99.5% of the AUC (area under the curve) of the full fitted model using the subset of 12 variables.

**Table 1:** Percent contribution, permutation importance and gain for the environmental variables included in the model.

Variable	Percent contribution	Permutation importance	Training gain without	Training gain with only
Mean temperature of driest quarter (BIO9)	28.3 (1)	2.1 (10)	99.8% (12)	58.9% (2)
Annual mean temperature (BIO1)	21.1 (3)	43.0 (1)	94.9% (7)	71.1% (1)
Slope	19.9 (2)	5.5 (8)	95% (1)	34.4% (4)
Temperature annual range (BIO7)	9.3 (4)	9.2 (5)	97.9% (6)	27.6% (7)
Precipitation of wettest quarter (BIO16)	7.3 (5)	17.3 (2)	96.4% (2)	9.4% (12)
Precipitation of warmest quarter (BIO18)	7.0 (10)	7.3 (3)	98.4% (4)	43.1% (3)
Precipitation seasonality (BIO15)	4.4 (7)	11.4 (4)	96.9% (5)	16.1% (10)
Isothermality (BIO3)	2.7 (9)	4.2 (7)	97.2% (3)	30.6% (5)

In parenthesis the rank of the variable contribution/importance in the model fitted with the whole set of 12 variables. Training gain values correspond to ratios relative to the full model.

A summary description of each variable used in the MaxEnt model (current values), as well as their projections for past and future scenarios, is shown in Supplementary Fig. S1. In the study area, all scenarios predict a shift towards an increase of annual mean temperature (BIO1) and mean temperature of the driest quarter (BIO9), and a decrease of precipitation of the wettest quarter (BIO16), from past to future. The temperature annual range (BIO7) had a reduction from MH to nowadays. The *Arbutus unedo* occurrence corresponds to a narrow range of annual mean temperature (BIO1) and mean temperature of the driest quarter (BIO9). The same pattern is visible in a smaller degree for isothermality (BIO3) and precipitation of the warmest quarter (BIO18). This suggests that the *A. unedo* is particularly sensitive to changes in those environmental variables.

In addition, we considered for each pixel the difference between the variable predicted value under a future scenario and its current value. The distribution of those differences is analyzed in the Supplementary Fig. S2. It shows how each climate variable is expected to change for the whole study area (solid lines) and for the occurrence locations (dashed lines). The BIO1, BIO7, and BIO9 values tend to increase over time, but only BIO1 increases consistently across the study area. The isothermality (BIO3) displays an approximately symmetric distribution, with changes up to 4 °C, according to the most severe scenario. However, in more than 90% of the occurrence sites, a decrease in BIO3 is predicted by the RCP 8.5 scenario, for both periods. There is an overall temporal trend decrease for BIO15, BIO16, and BIO18. Although an overall decreasing trend in precipitation seasonality (BIO15) is found over the study area, most occurrence sites will experience an increase, which suggests that locations where *A. unedo* is currently found will likely have more unstable precipitation conditions.

**Figure 1:**
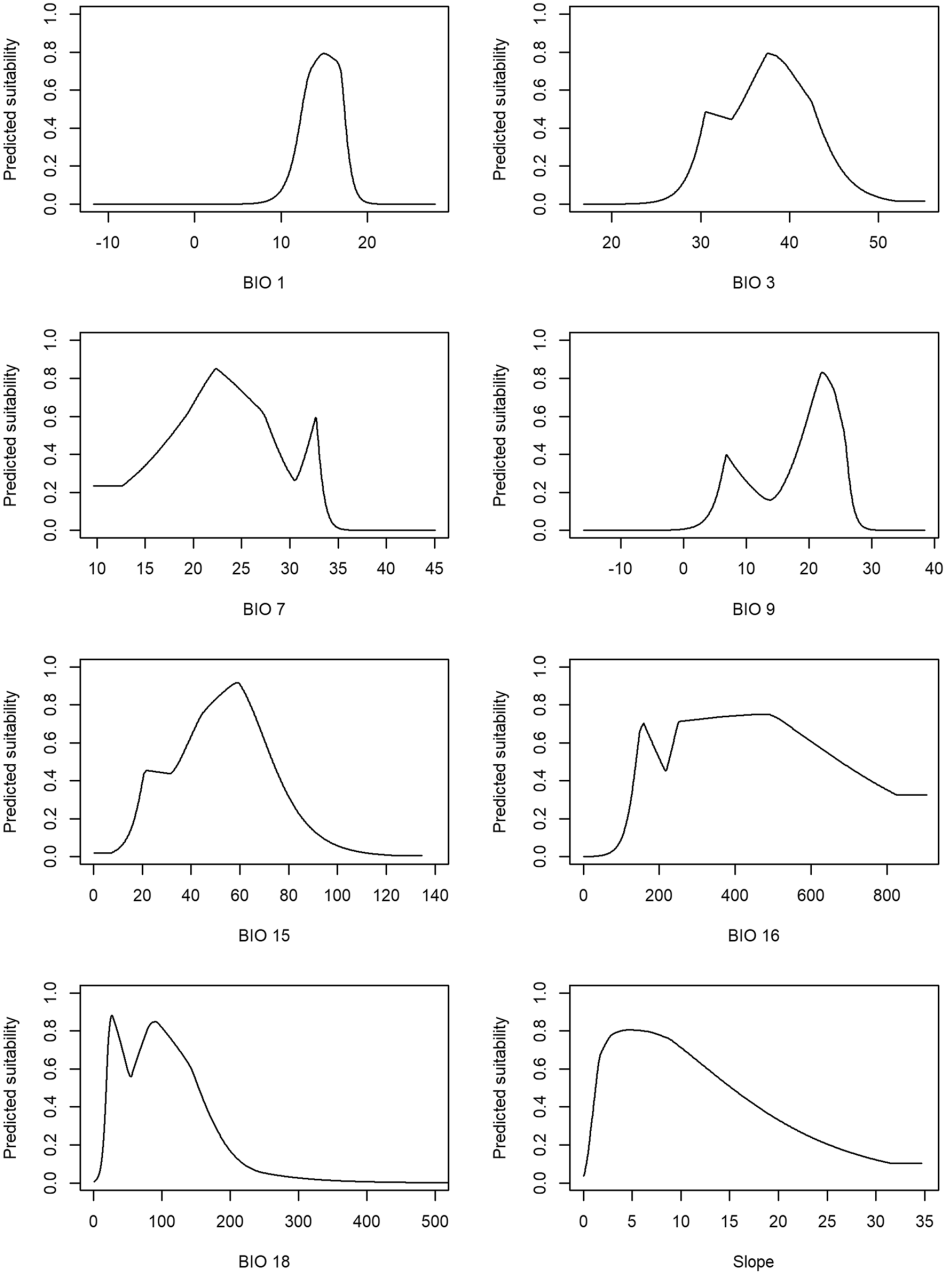
The response curves of predicted suitability values for the selected environmental variables. BIO1 (annual mean temperature, ºC), BIO3 (isothermality, %), BIO7 (temperature annual range, ºC), BIO9 (mean temperature of driest quarter, ºC), BIO15 (precipitation seasonality, %), BIO16 (precipitation of wettest quarter, mm), BIO18 (precipitation of warmest quarter, mm) and Slope (degrees).

Figure 1 depicts the response curves of predicted suitability values for the selected environmental variables and showed how predicted suitability is affected by each variable independently. In particular, this figure suggests that high suitability (> 0.6) is associated to the mean temperature of the driest quarter (BIO9) between 20 and 28ºC, the annual mean temperature (BIO1) between 12 and 18ºC, and the slope above 12 degrees. These results follow the patterns displayed in Supplementary Fig. S1 for the same variables.

### Potential past, current, and future suitability distributions

The current model was fitted using the occurrence records and the eight environmental variables previously selected, afterward used to predict suitability geographic distributions for two paleoclimates (LGM and MH) and four future climate scenarios (RCP 4.5 2050, RCP 4.5 2070, RCP 8.5 2050, and RCP 8.5 2070). The current model was statistically more robust than the random one (AUC = 0.5), with an AUC of 0.92, indicating the model's good performance^31^.

**Figure 2:**
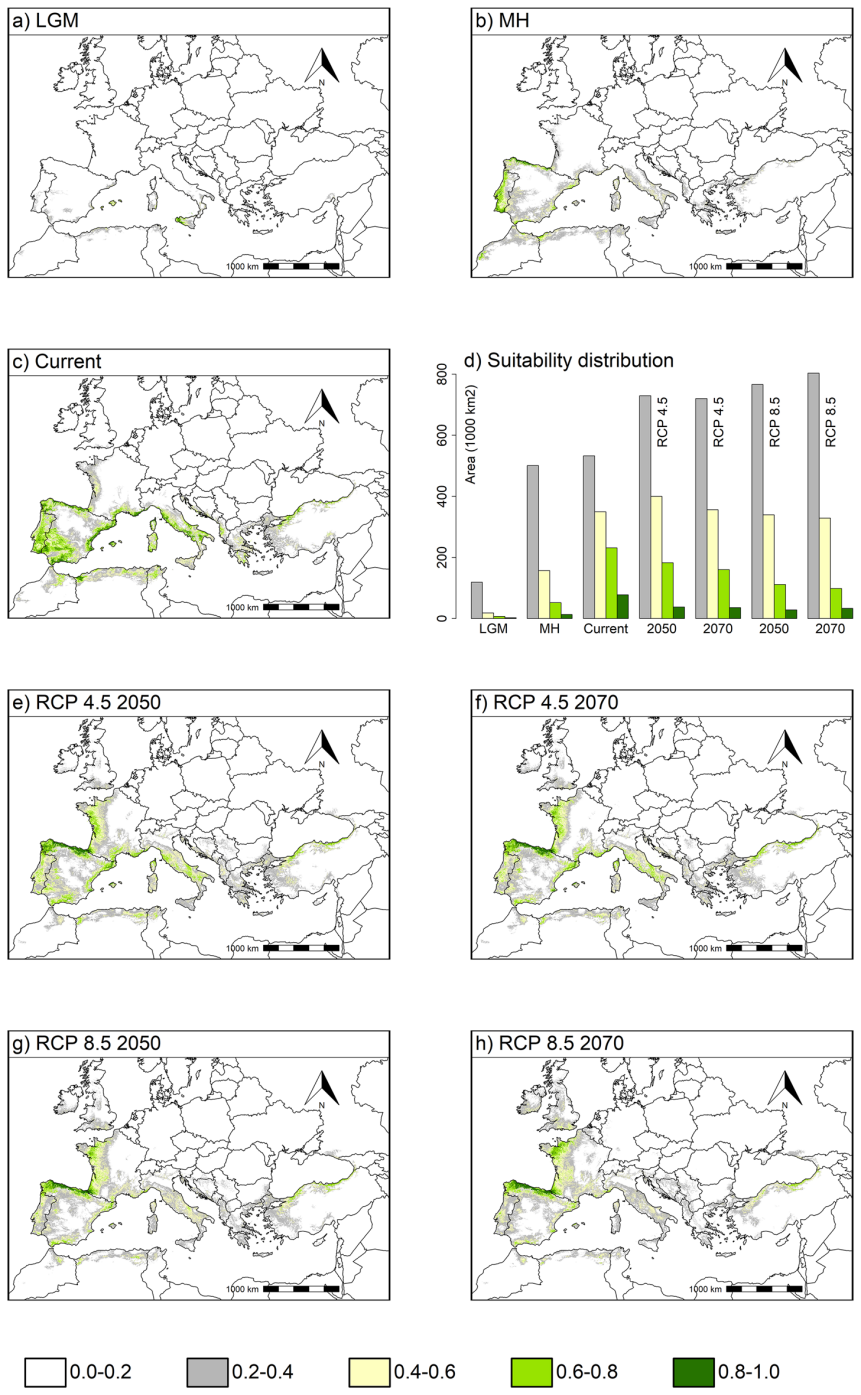
*Arbutus unedo* suitability areas. (**a**) LGM. (**b**) MH. (**c**) Current. (**d**) Evolution of the suitability areas from the LGM to the RCP 8.5 2070. (**e**) RCP 4.5 2050. (**f**) RCP 4.5 2070. (**g**) RCP 8.5 2050. (**h**) RCP 8.5 2070. Suitability class range: 0.0–0.2, non-suitable area; 0.2–0.4, low-suitability area; 0.4–0.6, general-suitability area; 0.6–0.8, medium-suitability area and 0.8–1.0, high-suitability area. Equal-area projection EPSG:3035. The map was made using the* sf *1.0–3 and the terra 1.4–14 (https://rspatial.org/terra/) packages in R 4.1.0^32,33^.

The past (LGM and MH), current, and future *A. unedo* potential distribution, under the global climate change scenarios RCP 4.5 and RCP 8.5, for the 2050s and the 2070s are displayed in Fig. 2. Suitability values (0 to 1) were classified into 5 classes (unsuitable to highly suitable). The MH environmental conditions were more suitable for the species development than the observed for the LGM, thus allowing a geographic expansion for the *A. unedo*, with a significant increase to the current climatic conditions. In the LGM, according to the fitted MaxEnt model, the species occurred in the Mediterranean islands (Sicily, Sardinia, and Balearic), East of Spain, South of Italy, and North of Africa.

In general, suitability increased from LGM to MH. Suitable conditions expanded to the South of Portugal, the North of Spain, and the North of Africa (Morocco and Algeria). The model for the present time exhibits the highest suitability for the *A. unedo’s* native range, meaning the Mediterranean Basin, overlapping the occurrences in the Iberian Peninsula, France, Italy, Croatia, Albania, Greece, Turkey, and North of Africa.

The projected suitability distribution for the future scenarios revealed new regions of medium to high suitability in the westernmost part of France, the south of England, and Ireland together with a reduction in the species’ potential distribution in the south of the Iberian Peninsula, Italy, and North of Africa. Figure 2d indicates an increase in the total area (km^2^) for low to high suitable areas (i.e., above 0.2), from the LGM to the present. An inverse trend occurs from the present time to future scenarios, more pronounced for the RCP 8.5.

**Figure 3:**
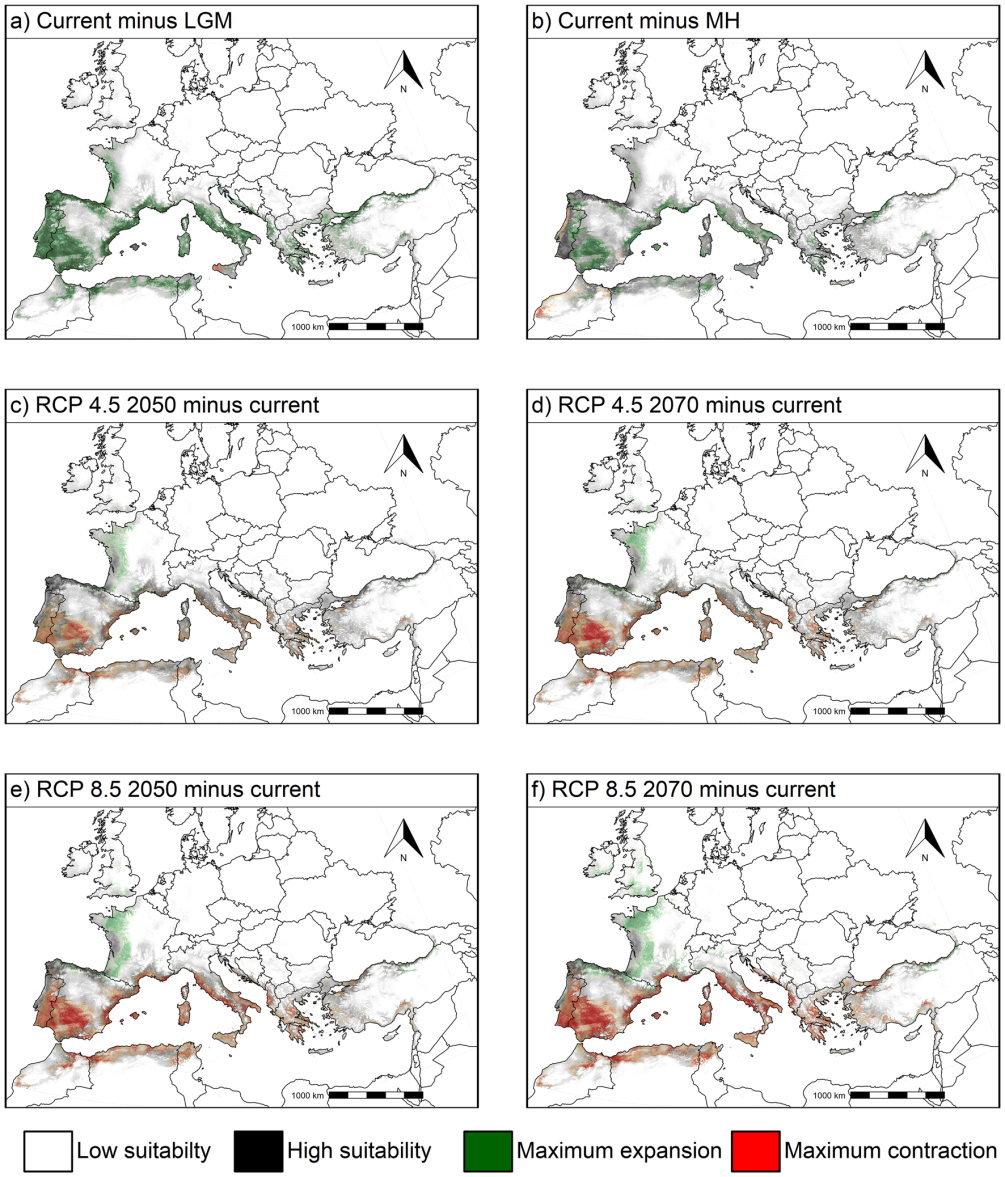
Changes in *A. unedo* distribution area between scenarios. Areas of expansion in green, and areas of contraction in red. The background grey intensity ranges from low current suitability (white) up to high current suitability (dark grey). Therefore, the four extreme cases are bright green (maximum expansion from low suitability); bright red (maximum contraction from high suitability); dark grey (suitability is and remains high) and light grey (suitability is and remains low). Equal-area projection EPSG:3035. The map was made using the *sf* 1.0–3 and the *terra* 1.4–14 (https://rspatial.org/terra/) packages in R 4.1.0^32,33^.

For each grid cell and each scenario, we computed the change in suitability relative to current one, to have a better picture about the *A. unedo’s* geographic distribution patterns evolution and predicted migration paths. Those suitability changes are shown in Fig. 3, stressing regions where contraction (reduction of suitability along time) and expansion (an increase of suitability along time) occurs. The range of possibilities for suitability changes covers four very distinct situations: large expansion from low suitability, large contraction from high suitability, constant high suitability, and constant low suitability.

**Figure 4:**
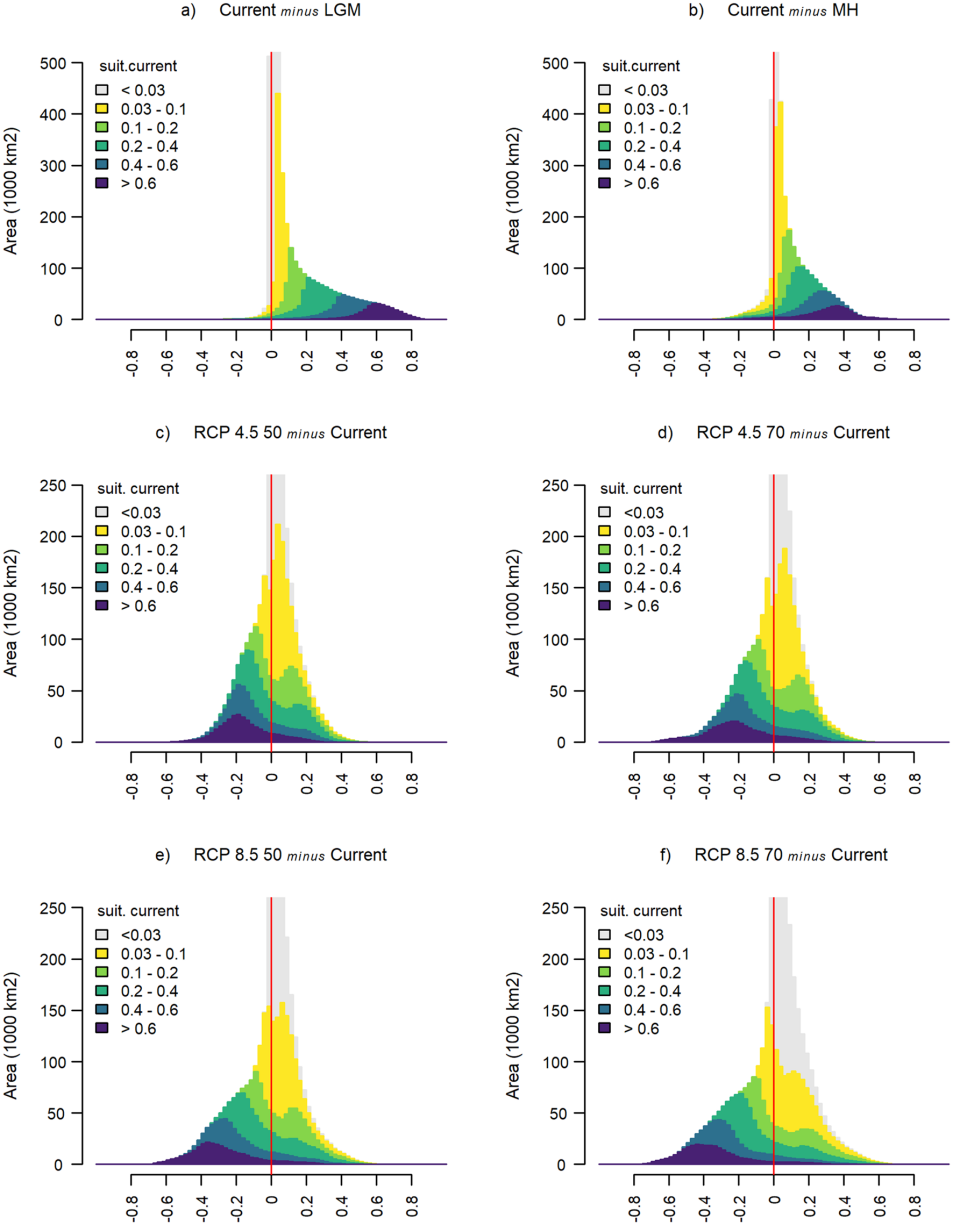
Differences between current and projected past and future scenarios suitability histograms. The x-axis is suitability_current_ – suitability_past_ for past scenarios and suitability_future_ – suitability_current_ for future scenarios. Each coloured band refers to a class of current suitability and its area in the histogram is proportional to the geographical area of the region with current suitability within this class. Areas with increasing/decreasing suitability (“expansion”/”contraction”) correspond to positive/negative differences and lies on the right/left hand side of the vertical red line. The area of each coloured band is the same across all histograms; the relation between the areas on each side of the vertical red line gives information about the extent of contraction relative to expansion in each projected scenario.

To quantify the total predicted areas of contraction and expansion for the *A. unedo*, we determined the distributions of suitability changes (Fig. 4), and the areas (in 1,000 km^2^), where contraction and expansion occurred (Table 2). Moreover, Fig. 4 and Table 2 also disaggregate total contraction and expansion areas into contributions from each class of current suitability.

**Table 2:** Area (10^3^ km^2^) of the regions where suitability is predicted to decrease (C, for contraction) and increase (E, for expansion) for each scenario.

Scenario		Sum area C/E	Current suitability				
			0.03—0.1	0.1—0.2	0.2—0.4	0.4—0.6	> 0.6
			C/E	C/E	C/E	C/E	C/E
Past	LGM	70/**2441**	28/**242**	17/**431**	16/**517**	8/**342**	1/**309**
	MH	266/**2243**	120/**750**	65/**382**	43/**490**	20/**329**	18/**292**
Future	RCP 4.5 50	1154/**1356**	196/**675**	157/**290**	**291**/242	**250**/99	**260**/50
	RCP 4.5 70	1208/**1303**	206/**664**	171/**277**	**305**/228	**259**/91	**267**/43
	RCP 8.5 50	**1295**/1215	228/**642**	175/**273**	**332**/201	**280**/70	**280**/29
	RCP 8.5 70	**1413**/1097	264/**606**	202/**246**	**365**/168	**296**/53	**286**/24

For past scenarios C corresponds to suitability_past_ > suitability_current_ and for future scenarios C corresponds to suitability_current_ > suitability_future_. Each column in the table contains the pair C/E for a class of current suitability and corresponds to a coloured band in Fig. 4, where C is the area on the left hand side of the red line and E is the area on the right hand side. In each cell, the largest value is highlighted in bold.

Figure 4 shows that for low current suitability (up to 0.1), the distribution of suitability changes is reasonably symmetric around 0, although a trend towards expansion in the future is visible for both RCP 4.5 and RCP 8.5. However, higher current suitability (darker colors in panels c to f) leads to larger areas, where a reduction in suitability is expected in the future (illustrated by negative skewness in Fig. 4). This is especially evident in the RCP 8.5 scenario. Indeed, as current suitability increased, the distribution becomes more asymmetric towards negative suitability changes, with this pattern being more pronounced for RCP 8.5 than for RCP 4.5. For high current suitability (above 0.6), there is a clear expansion from the past to the present and a clear contraction from the present to the future.

The areas reported in Table 2 do not include regions with current suitability below 0.03, since those broad regions are geographically distant from the species’ native distribution range, as shown in Supplementary Fig. S3. Table 2 revealed that there was always expansion from past scenarios to the present, regardless the present observed suitability. Overall, there was a net expansion of 2.37 million km^2^ since the LGM and 1.98 million km^2^ since the MH. For future scenarios, a net expansion in the RCP 4.5 2050 of 202,000 km^2^ is expected, but only 95,000 km^2^ in the RCP 4.5 2070, meaning a predicted contraction of 107,000 km^2^ from 2050 to 2070. Concerning the RCP 8.5 predicted scenario, the estimated net contraction for 2050 is 80,000 km^2^ and 316,000 km^2^ for 2070: summing up 236,000 km^2^ net contraction from 2050 to 2070.

**Figure 5:**
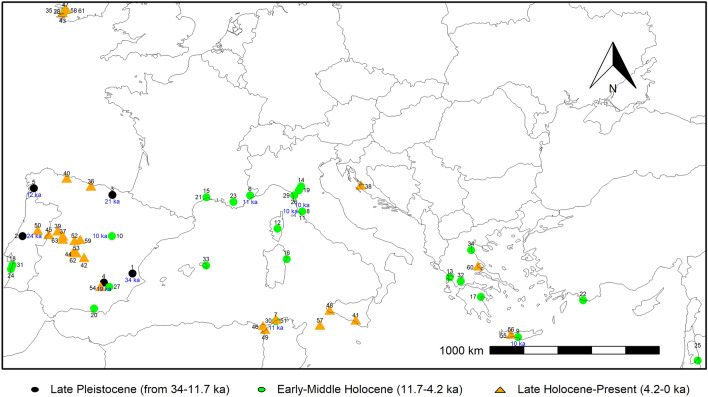
Map of the pollen sites in the Mediterranean region with *Arbutus/Arbutus unedo* records. Data retrieved from the Neotoma Paleoecology Database^37^. The site number 2 is a charcoal remain^46^. According to the oldest age of the record, the following site labeling was used: Late Pleistocene (from 34–11.7 ka), black-filled circles, Early-Middle Holocene (11.7–4.2 ka), green-filled circles, and Late Holocene-Present (4.2–0 ka), orange-filled triangles. The sites were numbered according to Supplementary Table S3, and the records age above 10 ka, for clarity, were displayed in deep blue. Equal-area projection EPSG:3035. The map was made using the *sf* 1.0–3 and the *terra* 1.4–14 (https://rspatial.org/terra/) packages in R 4.1.0^32,33^.

The fossil remains (Fig. 5, Supplementary Table S3), showed very few pieces of evidence from the Late Pleistocene, all of which in the IP, with the oldest record dating from 34 ka (site number 1) in the Valencia region. This might not indicate the species’ absence in previous ages, but owing to entomophile pollination and being a low pollen producer, it tended to be under-represented^34,35^. Additionally, the pollen is large and heavy and does not travel far from the tree, thus it will not be found in large quantities, e.g., in lake sediment cores^36^. Besides, it is similar to some other pollen from the same family, and, probably, not distinguished in some investigations, and possibly included in the Ericaceae family. The Late Pleistocene-Early Holocene remains were mostly found nearby the sea, usually in the IP. Most of the sites were found in the westernmost part of the Mediterranean Basin, and the southern presence was found in north-western Tunisia, Sicily, and Crete. The easternmost presences were found in south-western Turkey and the Dead Sea. Caution about the inferences made from pollen in the easternmost part of the Basin must be observed since, in that region, the *A. andrachne* range overlapped the *A. unedo*^23^. Many Late Holocene records were found inland in the IP. The pollen grains and macro remain records indicated that the species was present before the Last Glaciation in the region, and the information showed a more intense signal in the Basin westernmost region, in particular the IP, which points out to preferential refugia areas therein. Nowadays, the species density is more intense in this region (Fig. 2c). Almost all the remains were found nearby the sea, which indicates that the presence of water was key for ice ages’ drawback attenuation, but also in the preferred current distribution, indicating that the species is drought resistant, yet with limitations.

## Discussion

### Current and past scenarios

The *Arbutus unedo* distribution presence database and eight environmental variables were used to predict the species potential distribution under past, current, and future climatic conditions. The current fitted model predicted the *A. unedo* suitable habitat with good performance, when evaluated using AUC (AUC = 0.92)^31^.

The results suggest that the species distribution was mainly determined by the following attributes: (1) mean temperature of the driest quarter (BIO9), (2) annual mean temperature (BIO1), (3) slope and (4) temperature annual range (BIO7), summing up to 78.6% of the model’s total contributions (Table 1). In a recent study for the same species in Portugal, the authors considered that the variables precipitation seasonality (BIO15) and slope had a net significant influence on the species’ habitat suitability^16^. The variable slope was expected to influence the *A. unedo* distribution, since this tree is ecologically adapted to rocky slopes, which are edaphically unsuitable for woodland, where the vegetation is limited to shrub communities. The *Arbutus unedo* is particularly intolerant to shade, and this high light requirement is particularly needed for fruit production, restricting this species to settlement with open habitats^21^. This aspect was confirmed in a study about a woodland disturbance, where the *A. unedo* widespread initially, and, afterward, the species declined as canopy woodland re-developed^35^.

**Figure 6:**
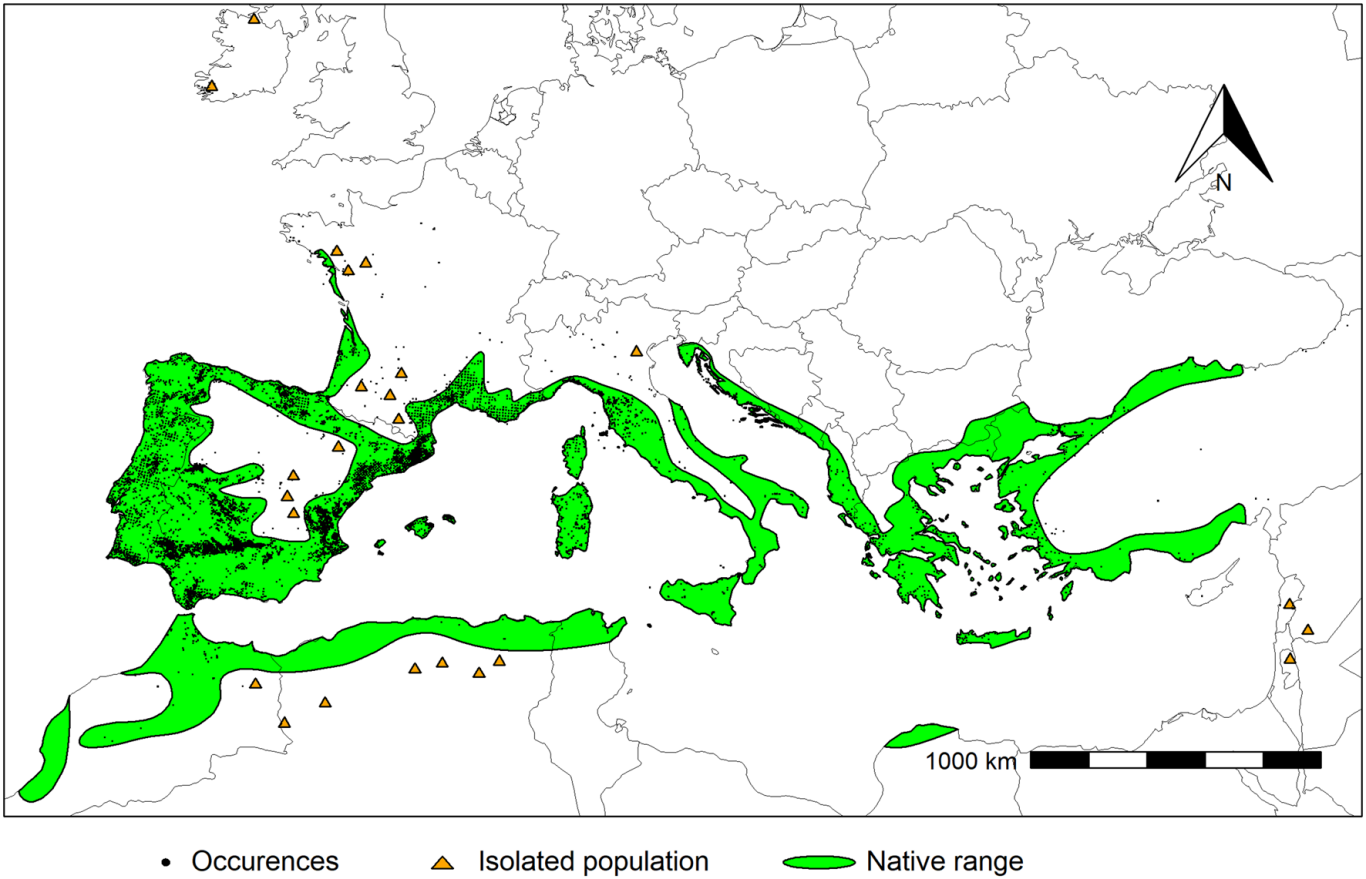
*Arbutus unedo* L. distribution area and occurrences. Black dots represent the species’ occurrences (Supplementary Table S1), and the green area represents the species native distribution range for *A. unedo* downloaded from the data sets in^38^. Equal-area projection EPSG:3035. The map was made using the *sf* 1.0–3 and the *terra* 1.4–14 (https://rspatial.org/terra/) packages in R 4.1.0^32,33^.

The *Arbutus unedo* was adapted to a wide range of climatic conditions considering the response curves analysis (Fig. 1). According to Santiso^24^, this species developed a conservative strategy in the use of nutrients and water, when they are scarce. The *Arbutus unedo* plasticity and evolvability explained the current species persistence throughout its distribution range, which will be decisive in future response to climate change. The species distribution prediction for the present is in agreement with the species distribution according to Caudullo et al.^38^ (Fig. 6). Indeed, the *A. unedo* is widespread in Portugal, Galicia, and southwest of Spain, occupying the coastal belt from Tunisia to Morocco along the North of Africa, and from the Iberian Peninsula to Turkey across southern Europe, yet occurring in the northern Iberian Peninsula, western France, and south-western Ireland. The results showed that MaxEnt fitted model suitability prediction was high in those regions, which was further supported by the high observed AUC. Furthermore, it is important to stress the current potential distribution meaningful accuracy, and, therefore, the ability to extrapolate past and future prediction scenarios for *A. unedo* distribution in the Mediterranean Basin. Not surprisingly, the highest predicted suitability for the species occurs mostly in its native range, in regions where the mean January temperature is above 4ºC, a limit required for the species’ survival^23^. Unfortunately, this variable is only available in the WorldClim database for the present, but not for the future or past periods. Nevertheless, the species’ current spatial distribution was overlapped to the areas with mean January temperature higher than 4ºC, and a high overlap is reckoned, thus confirming this factor ecological importance^17,23^. The analysis of the species' predicted suitability show that high-suitability areas [0.8–1.0] are more frequent in current conditions, compared to other scenarios, and thus confirming the suitability of climate current conditions for the species’ development (Fig. 2d).

The predicted habitat suitability for the LGM and MH scenarios differed, as expected, from the obtained for the current climatic conditions. The climatic conditions in the MH, were more suitable than during the LGM, due to climate warming, thus allowing the species expansion. According to the LGM projections, the species occurred in the Mediterranean islands (Sicily, Sardinia, and Balearic), North of Italy, south and eastern coast of Spain, Catalonia, North Africa, and spots in Portugal, Greece, and Turkey (Fig. 2a). These were suitable putative cryptic refugia areas that remained during the LGM. Keppel et al.^39^ defined refugia as locations to which species retreated during periods of adverse climate, and could potentially expand from, when environmental conditions turn out to be more suitable for the species. These locations/habitats were responsible for the species’ survival under changing environmental conditions for millennia. Identifying and characterizing climate refugia provides an important context for understanding the modern species distribution development, traits, and local adaptation^40^. The Iberian, Italian and Balkan peninsulas, which remained relatively ice-free during the ice ages were identified as the main glacial tree refugia areas in Europe^41,42^.

The Betic mountains, in the Iberian Peninsula (IP), were sought to be a species’ refuge from the last ice age, nearby the sea (Valencia region, Spain), where fossil pollen pieces of evidence were found in the Canal de Navarrés peat deposit^4^, with 34 ka, and in the Siles lake (ca. 19 ka) located in the Segura mountains of southern Spain^43^ (Fig. 5, numbers 1 and 4, respectively). These mountains were ice-free during the Late Pleistocene, and the persistence of a mild climate could explain that other thermophilic species have prevailed in this particular region during the Full Glacial, proved by high genetic diversity levels^44^, and with the pollen spectra suggesting the region as a glacial refugium for temperate and Mediterranean trees^9,13,43^.

The Iberian Peninsula border could have been a refuge for the species, due to the importance of the sea as a temperature regulator. Indeed, pollen evidence was found in the Basque mountains (northern IP, Fig. 5, number 3: 21 ka)^45^. Despite the information given by the MaxEnt modelling, the *A. unedo* reconstruction for the LGM was observed in other areas, since paleo-evidences were found in unexpected northern sites (Donatella Magri, personal communication). Certainly, fossil charcoal pieces of evidence were found in the central region of Portugal (Serra do Sicó), nearby the sea, supporting the presence of thermophilous taxa, including *A. unedo*, during the Full Glacial (24 ka, Fig. 5, number 2), and the presence of other Mediterranean taxa, such as olive tree and *Pistacia lentiscus* L., suggested that this may have been refuge zones for thermophilous plants^46^. Corroboration of the species expansion later during the warm and humid period of the Holocene (see Fig. 3b; green area) was confirmed by pollen remains (Fig. 5), particularly inland and northwardly, in the IP, and, also, in a molecular population genetics study made in Portugal, with northward haplotype migration^47^.

Santiso et al.^17^ in a study about the *A. unedo* phylogeography, concluded that this species had two clades, separated during the late Pleistocene, before the LGM, suggesting that it may have coincided with the hardest glaciations recorded in the Quaternary. One clade occupied the Atlantic Iberia and, possibly, North Africa, while the other occurred in the western Mediterranean Basin in Spain. Besides, the *A. unedo* possibly persisted in the late Quaternary in the western Mediterranean, based on chloroplast DNA observations, and the results from the current study supported this interpretation, and by the fossil evidence (Supplementary Table 3). The same strong genetic differentiation between the western and eastern Mediterranean Basin was also found in olive lineages, and this pattern is congruent in other Mediterranean shrubs and tree species^9,13,15^. In another species (maritime pine), Bucci et al.^44^ confirmed the existence of a genetic divide between eastern and western lineages, also previously described by Burban and Petit^48^ based on mitochondrial DNA. Additionally, maximum haplotypic diversity for this species was found in south-eastern and central Spain, which, therefore, may be considered a biodiversity hotspot and a strong signal for refuge, as long-term populations tend to harbour more diversity than recently expanding ones^41^. In their study with *A. unedo* in Portugal using cpSSR, Ribeiro et al.^47^ found signals of two putative refugia in southern and central littoral in the country, also supported by macrofossil and pollen remains. Furthermore, according to Médail and Diadema^41^, several regions in the Western Mediterranean (large Mediterranean islands, North Africa and Catalonia), could have played a role in the case of *A. unedo*, and sought as refugia locations.

The *Arbutus unedo* suitability maps obtained for past conditions support the claim that MH climatic conditions were more suitable for the species expansion than the LGM ones (Fig. 2a,b, and d). Consequently, spatial distribution changes analysis between past and current scenarios (Fig. 3) showed that *A. unedo* potential distribution expanded extensively from the LGM-current and the MH-current periods, indicating that more suitable areas were available for the species at present. Pollen records during the Early-Middle Holocene supported the Mediterranean presence of this species, in particular, nearby the sea (Fig. 5), which is under the MaxEnt predicted eastward dispersion (Fig. 3a, b). In another species bird-dispersed, the *Myrtus communis* L. (myrtle), a spread from west to east was verified, following genetic differentiation during the Pleistocene^15^, since the western region of the Mediterranean Basin had a milder climate, compared to the eastern one, during the LGM.

The *Arbutus unedo* had, probably, a considerable ability to disperse, migrating over thousands of kilometres and even crossing sea stretches, allowing the species expansion during the MH, when the climatic conditions became more favourable^17^. In the future, *A. unedo* migration possibility will depend on climatic change pace and, also, on seed dispersal, particularly long-distance dispersal events^49^, since long-distance migration fitness will be useful in future change scenarios, allowing species to progress to newly suitable areas, as happened in the past. Nevertheless, from past evidence in Ireland, Britain, and across Europe, trees migrated faster than would be expected, during the warming period at the beginning of the Holocene, and that biotic and/or abiotic vectors must have been involved in dispersal^50^. This is the case with *A. unedo*, the seeds are dispersed by different type of birds that eat the soft fruit and, some of them are migratory species, as the European robin (*Erithacus rubecula* L.)^51^.

### Future scenarios

The impact of climate change on the *A. unedo* potential distribution was assessed under two representative concentration pathways (RCP 4.5 and RCP8.5) for the years 2050 and 2070. The results showed that climate change, under both moderate and high emission scenarios, will affect the species distribution range. A decrease in the predicted suitable areas was, generally, observed, since the climate becomes less favourable for the species in the future.

According to the results, under the RCP 4.5 scenario, the potential distribution area will increase up to 2050, and decrease afterward according to the prediction for 2070 (Fig. 2d, e, f). Considering the RCP 8.5 scenario, the suitable area will exhibit a net decrease from the present up to 2050 and continue this trend until 2070. It is also expected that he medium–high suitable areas will gradually decrease from the present to the future, especially for scenario RCP 8.5. These results are in agreement with those obtained by other authors, confirming that Mediterranean species (e.g. *Quercus* sp.) distribution areas will be negatively affected by future climate change^52,53^.

Several studies concluded that global warming will influence species distributions by causing expansions, contractions, or shifts in the species ranges^2^. As expected, the results from the current study showed that suitable areas will contract under future climate scenarios when compared to the current conditions, though suitable areas will emerge. These effects' impact will depend on the climate change scenario severity. Despite contraction and expansion effects, the presence of the species will gradually decrease from the 2050s to the 2070s. Moreover, a species’ shift toward the North will be verified, because of suitable areas emergence, observed mainly in the RCP 8.5 scenario. Those areas will mostly emerge in the North of France, South of the United Kingdom, and Ireland, implying species’ latitudinal migration. The species’ northward displacement is consistent with climate change studies' results obtained by several authors, including *A. unedo*^16,20,54,55^. Nevertheless, according to Gerassis et al.^20^, under climate change, the expected habitat disruption and fragmentation could lead to very adverse conditions for *A. unedo* survival in the future, which could undoubtedly conduct to a possible species’ presence decline in most of the current distribution area. Moreover, distribution models that predict climate‐induced range shifts do not account for spatial dispersal variation^56^, but adaptive dispersal evolution always reduced neutral genetic diversity across the species' range. This means that the species’ genetic pool might be erased, depending on the climate change velocity, amongst other conditions, like landscape fragmentation and competition with other species/crops^16,57^. Additionally, the species ability to migrate mainly through seeds, dispersed by migratory frugivorous birds, bird abundance, and the velocity of climate change, are key issues for future species survival^23,58^.

These results suggested that future changes in environmental conditions may lead to suitable habitat loss in areas where the species had persisted and with a possible range shift towards the North. These findings also revealed that with continuous future climate warmth, the current potential distribution *A. unedo* areas will become unsuitable or contract, leading to significant changes in the species' current distribution pattern and putative presence loss. The possibility of species’ migration will ultimately depend on its capability to keep pace with the changing conditions and the velocity to adapt to environmental changes, such as those presented by habitat and climate modifications^56^.

The Mediterranean Basin is one of the most vulnerable climate change hotspots in the world, thus understanding how future climate changes will disturb Mediterranean plant species distribution will be key for tree management planning and conservation design. Nevertheless, further investigation is needed for species well adapted in this region to assess the impacts of climate change in their current and future potential distributions. Those studies including past climate impact on species distribution should be complemented with phylogeographic methods and paleoclimate reconstructions to locate refuges. Other species distribution models, besides MaxEnt, could be tested, although the lack of absence records data limits considerably the modelling approach. Additionally, the human influence magnitude on the predicted ecological niche should be further studied in detail, including urbanization, industrialization and putative tourism pressure, particularly in coastal areas.

## Material and methods

### Species occurrence data

The kick-off step consisted of building an extensive and up-to-date database for the presence of the native species, considering the previous information according to Caudullo et al.^38^. Georeferenced data of *A. unedo* occurrence was compiled from the literature and online databases (Supplementary Table S1). A preliminary coordinate system standardization and data cleaning were performed using the ArcGIS® Desktop 10.6. Occurrences with georeferencing errors were discarded, and duplicated records were removed. The resulting dataset is composed of 13,293 occurrences, represented as latitude/longitude coordinates in the WGS84 coordinate reference system.

The study area, with latitude between 30.92º N and 59.26º N, and longitude between 10.61º W and 42.16º E, was divided into a grid cell of ca. 1 km^2^, consistent with the environment variables spatial resolution, using ArcGIS. Finally, the geodatabase was reduced to one occurrence per unit cell to attenuate spatial autocorrelation^31^, summing up to 11,456 occurrences (Fig. 6).

### Environmental variables

Two types of environmental variables were used to calibrate the current model: 21 climatic variables—tmax, tmin and 19 bioclimatic variables (BIO1 to BIO19), extracted from the WorldClim 1.4—Global Climate database^59^, with a spatial resolution of 30 s (~ 1 km^2^), and two topographic variables—elevation and slope (for definition and units see Supplementary Table S2). The elevation data was obtained from the Digital Elevation Model (DEM) provided by the Global Multi-resolution Terrain Elevation Data 2010^60^, with a 30 s-resolution (ca. 1 km^2^). The slope, a morphometric variable, was computed with the ArcGIS Spatial Analyst extension.

Some of these variables are highly correlated and, therefore, appropriate dimensionality reduction is critical to discard spurious results and to optimize the final fitted model. From the original set of 23 variables, an exploratory data analysis was performed aiming at discarding the most correlated ones, retaining a subset that preserves a high proportion of the total variance. Function *eleaps*, with criterion ‘RM’, from the R package ‘Subselect’ (https://CRAN.R-project.org/package=subselect) was applied to the correlation matrix of a set of 100,000 data points randomly chosen from the background (by sampling a large number of points throughout the study area the background points or background was obtained), and the best 12 variables subset was selected. This set of variables was further reduced according to evaluation metrics of the species distribution model (details in the section below).

### MaxEnt modelling

The 12 selected variables' predictive ability was further explored using the MaxEnt version 3.4.1. The MaxEnt is a maximum entropy species distribution modelling software. This machine-learning software estimates a density within the space of environmental variables, searching for the most uniform distribution, subject to constraints imposed by the species’ observed occurrences^27,61^, and became the software of choice when presence-only data was available^62^. The MaxEnt algorithm input is a data matrix where columns correspond to the environmental variables and rows to all grid cells overlapping the study area, and where a subset of rows corresponds to the *A. unedo* occurrences. The MaxEnt uses the presence points and a background sample to fit a model over the space of the environmental attributes. Instead of simply considering the distribution for the original variables, the MaxEnt software explores those distributions in high-dimensional feature space, applying transformations to the original variables, and selects an optimal subset of those features. The considered output was the cloglog (complementary log–log)^63^ MaxEnt score, which ranges on [0,1] and is called “suitability”, with the higher values corresponding to a higher likelihood of the grid cell being a suitable location for the *A. unedo*. Different regularization values were tested to check the variables’ curves' smoothness, and the default regularization multiplier equal to 1 showed no considerable differences compared to other values. The MaxEnt parameters’ settings included removing duplicate presence records, using a regularization multiplier of 1, a maximum number of iterations of 2,500, and 100,000 background points. The remaining parameters were set to default values.

To determine the key variables for the MaxEnt model, the percent contribution, the permutation importance, and the Jackknife test results were considered. The former measures the accumulated gain contribution due to a given variable along the steps to fit the MaxEnt model. Therefore, it depends not just on the fitted model but also on the algorithm search path. Each variable permutation importance was calculated by permuting the values in the corresponding column in the input matrix and comparing MaxEnt’s gain before and after permutation. The most important variable was the one associated with the steepest decrease. Finally, Jackknife tests compared MaxEnt gain, either by removing the i-th environmental variable (“without”) or by using solely the i-th variable (“with only”) in the model. The Jackknife validation, the ratio between the gain of each submodel, “without” and “with only” and the gain of the full model, was considered. For the selected set of variables, the AUC — Area Under the Curve of the Receiving Operator Characteristics (ROC) — was used to assess the model’s accuracy. The AUC is a threshold for the independent measure of predictive accuracy based, only, on the ranking of occurrence,s and is interpreted as the probability that a random occurrence is ranked higher than a random background point^29^. The AUC ranges from 0 to 1, with higher values corresponding to better model’s prediction^31,64^. Furthermost, the AUC works not only as an indicator of the model accuracy but provides a criterion to select a subset of the MaxEnt’s inputted variables, consisting in a heuristic procedure driven by variable importance criteria applied to find the smallest submodel retaining at least 99.5% of the full model AUC. The MaxEnt model was fitted using the gathered extensive occurrence dataset over the space of the selected environmental variables for the near-current climate data. The future *A. unedo* distribution was then predicted for two climate change scenarios or Representative Concentration Pathways (RCPs): the RCP 4.5 (moderate emission scenario achieving an impact of 4.5 watts per square meter by 2100) and the RCP 8.5 (hard/extreme emission scenario accomplishing an increase of 8.5 watts per square meter by 2100)^30^. Future projections were computed for two time-slices: 2050 (average for 2041–2060) and 2070 (average for 2061–2080). The *Arbutus unedo* past distribution was predicted for the Mid-Holocene (MH, about 6,000 years ago), and the Last Glacial Maximum (LGM, about 22,000 years ago). For the past and future projections, we used the global circulation data model (GCM) — CCSM4 (Community Climate System Model 4.0) described in^14,16,52^ and available in WorldClim 1.4. The data spatial resolution used for the future and the MH was 30 s (ca. 1 km^2^). The LGM resolution was 2.5 min (ca. 5 km^2^), which implied the resampling of the LGM layers to 1 km^2^ resolution.

Many studies, e.g. Radosavljevic and Anderson^65^, rely on a threshold of suitability to distinguish “suitable” from “unsuitable” regions for the species. The choice of a threshold is questionable in the case of presence-only data, since species’ prevalence cannot be estimated, and MaxEnt scores cannot be interpreted as probabilities of presence^29,66^. Therefore, we analyze variations of suitability, across climate changes, avoiding using arbitrary thresholds. We note that this threshold-free approach is robust to all available transformations of scores in MaxEnt (e.g. cloglog and logistic). Although thresholding allows model validation based on error matrices, it introduces bias on error estimates^67^.

### Palaeobotanical data

Sixty-three sites corresponding to fossil 62 pollen records and 1 charcoal remain were found in literature and public databases^37^, and further compiled in Supplementary Table S3 (Fig. 5 ).

## Supplementary Information

Supplementary Information.

## Data Availability

The authors declare compliance with Scientific Reports’ policy regarding the data availability. All relevant data are within the paper and its Supplementary Information files, and any other information about this paper can be given upon request to the corresponding author.
