# Supplementary material for: Prediction scenarios of past, present, and future environmental suitability for the Mediterranean species Arbutus unedo L
Source: Sci Rep. 2022 Jan 7;12:84. doi: 10.1038/s41598-021-03996-0 (PMC8742115; doi:10.1038/s41598-021-03996-0)
Supplement: Supplementary file 1 — Supplementary Information. [file 41598_2021_3996_MOESM1_ESM.docx]

**Supplementary Information**

**Prediction scenarios of past, present, and future environmental suitability for the Mediterranean species *Arbutus unedo* L.**

Alice Maria Almeida^1,2^, Maria João Martins^3,4^, Manuel Lameiras Campagnolo^3,4^, Paulo Fernandez^1,5^, Teresa Albuquerque^1,6,7^, Saki Gerassis^8^, José Carlos Gonçalves^1,6,9^, Maria Margarida Ribeiro^1,3,6,9*^

^1^Instituto Politécnico de Castelo Branco, Castelo Branco. Portugal.

^2^C4 - Centro de Competências em Cloud Computing (C4-UBI), Universidade da Beira Interior, Covilhã, Portugal.

^3^Forest Research Centre, Instituto Superior de Agronomia, Tapada da Ajuda, Lisbon, Portugal.

^4^Departamento de Ciências e Engenharia de Biossistemas, Instituto Superior de Agronomia, Tapada da Ajuda, Lisbon, Portugal.

^5^MED – Mediterranean Institute for Agriculture, Environment and Development, Universidade de Évora, Évora, Portugal.

^6^Centro de Recursos Naturais, Ambiente e Sociedade (CERNAS) - Instituto Politécnico de Castelo Branco, Castelo Branco, Portugal.

^7^Instituto Ciências da Terra, Universidade de Évora, Largo dos Colegiais, Évora. Portugal.

^8^Department of Natural Resources and Environmental Engineering, Universidade de Vigo, Lagoas, Marcosende, Vigo, Spain.

^9^Centro de Biotecnologia de Plantas da Beira Interior. Quinta da Senhora de Mércules, Castelo Branco. Portugal.

**Contents**

Supplementary Figure S1. Distribution of each variable included in the model, across all the considered time periods: past (LGM, MH), current and future.

Supplementary Figure S2. Distribution of differences between variables in future and in present.

Supplementary Figure S3. Regions (in green) where current suitability is higher than 0.03.

Supplementary Table S1. Data gathering sources.

Supplementary Table S2. Original set of the 23 environmental variables (climatic and topographic) that were selected for modelling.

Supplementary Table S3. Summary of known fossil occurrences of *Arbutus* and *Arbutus unedo* from Pleistocene to present ranked by the age of the oldest record.

Supplementary References.


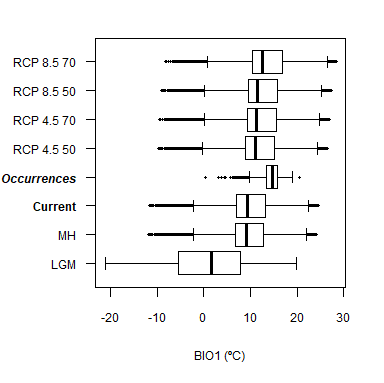

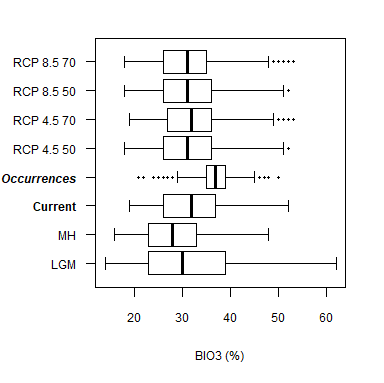

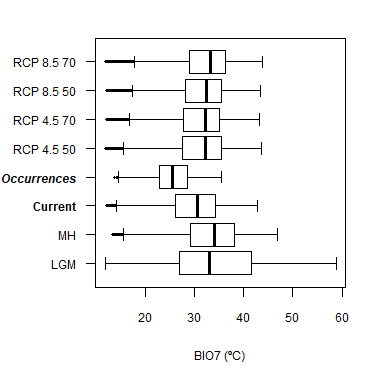

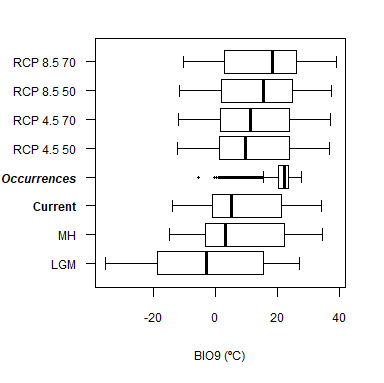

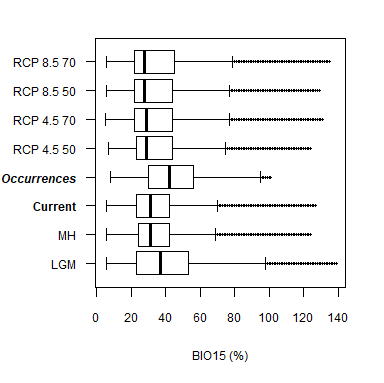

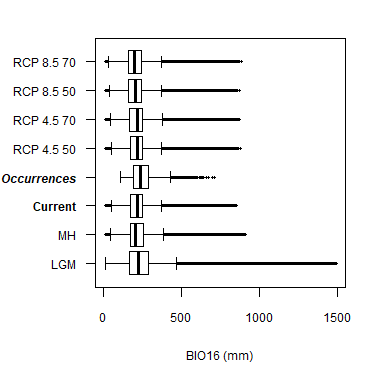

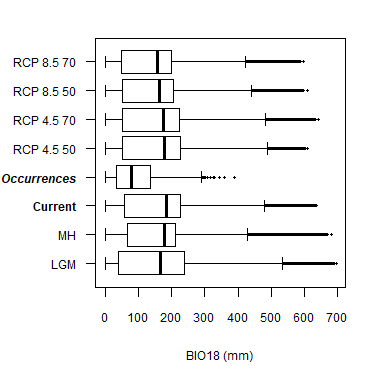

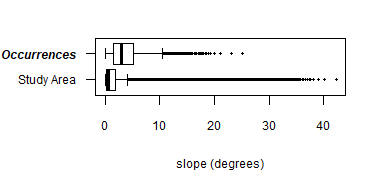


**Supplementary Figure S1.** Distribution of each variable included in the model, across all the considered time periods: past (LGM, MH), current and future.


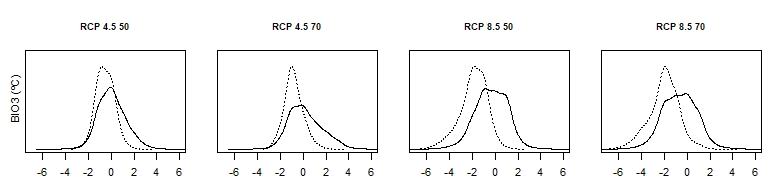


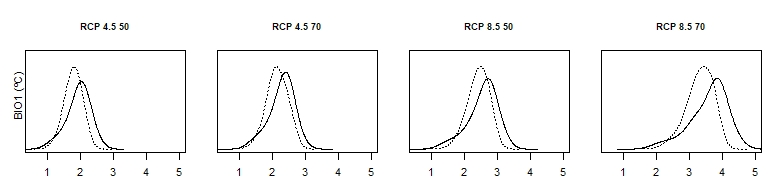


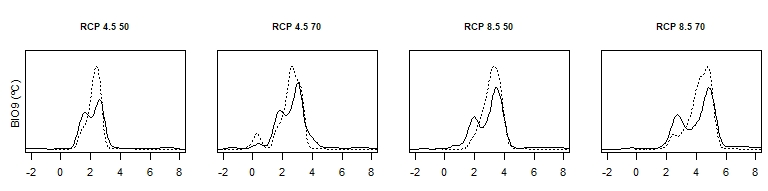


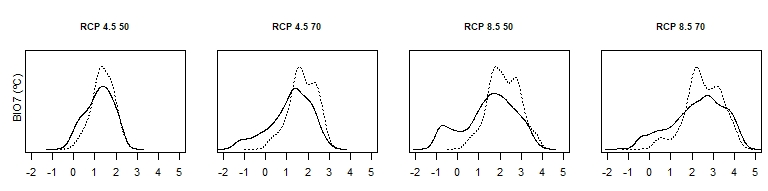


**Supplementary Figure S2.** Distribution of differences between variables in future and in present. Solid lines for the study area and dashed lines for the current occurrences. BIO1 = annual mean temperature; BIO3= Isothermality (BIO2/BIO7); BIO7 = temperature annual range (max. temperature of warmest month - min. temperature of coldest month); BIO9 = mean temperature of driest quarter; BIO15 = precipitation seasonality (coefficient of variation); BIO16 = precipitation of wettest quarter; BIO18 = precipitation of warmest quarter.


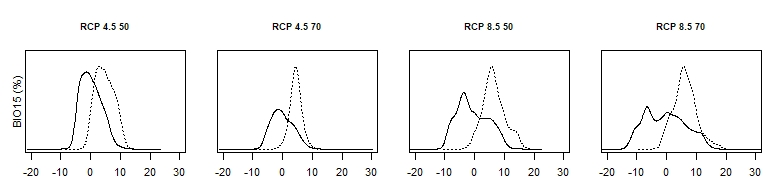

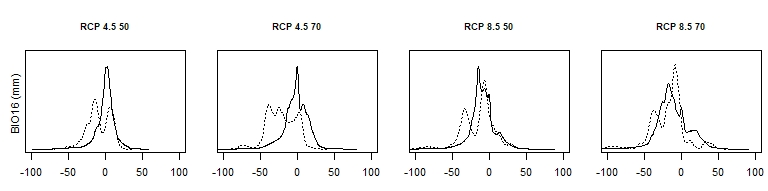


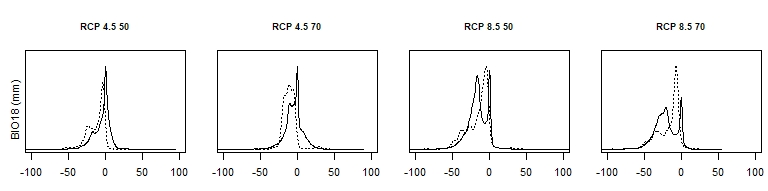


**Supplementary Figure S2.** (Continuation). Distribution of differences between variables in future and in present. Solid lines for the study area and dashed lines for the current occurrences. BIO1 = annual mean temperature; BIO3= Isothermality (BIO2/BIO7); BIO7 = temperature annual range (max. temperature of warmest month - min. temperature of coldest month); BIO9 = mean temperature of driest quarter; BIO15 = precipitation seasonality (coefficient of variation); BIO16 = precipitation of wettest quarter; BIO18 = precipitation of warmest quarter.


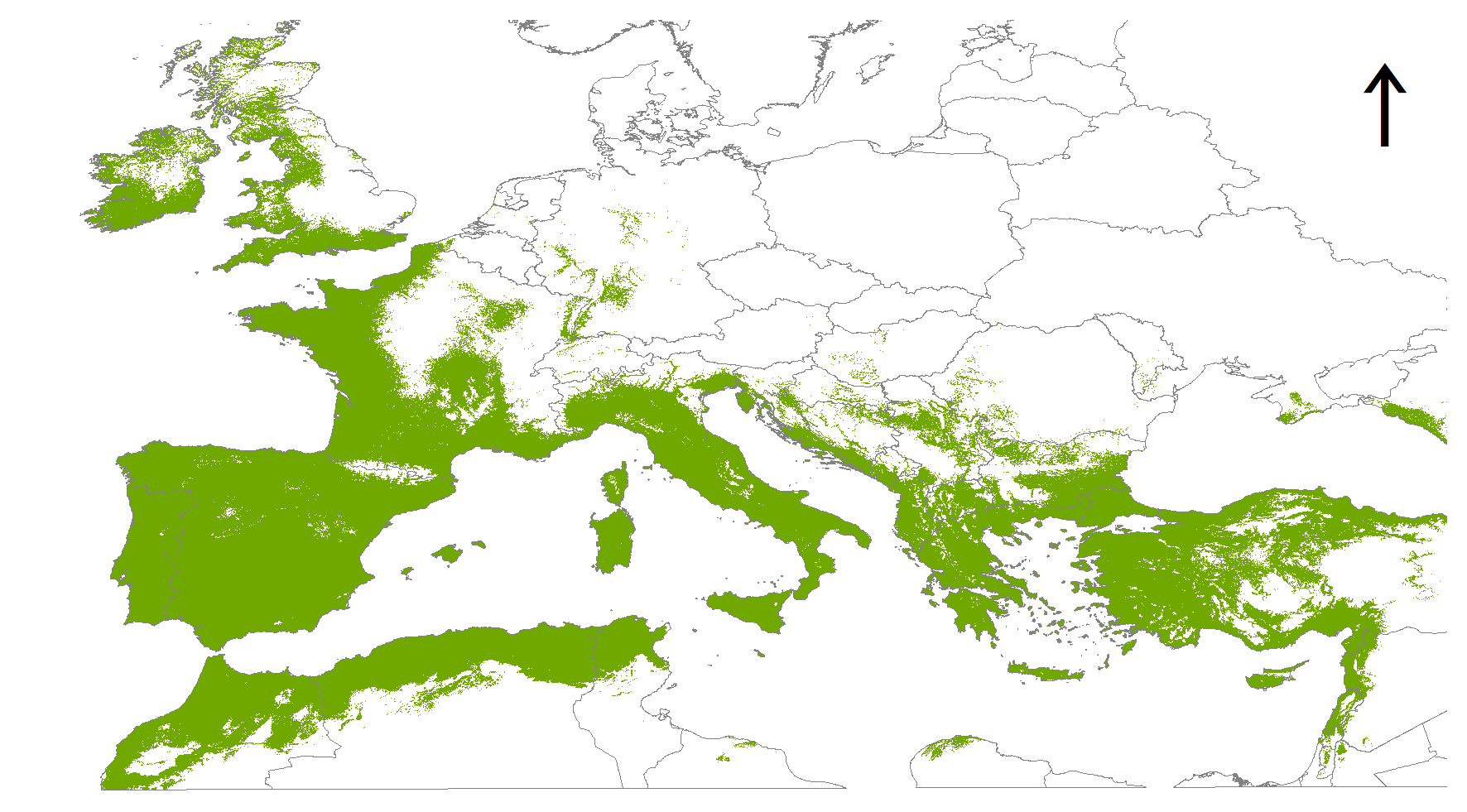


**Supplementary Figure S3.** Regions (in green) where current suitability is higher than 0.03. EPSG:4326. The map was built with ArcGIS® Desktop 10.6.

| 1. **Source/Reference** |
| --- |
| 1. GBIF \| https://www.gbif.org/ |
| 1. iNaturalist \| https://www.inaturalist.org/observations |
| 1. Flora Croatica Database \| https://hirc.botanic.hr/fcd/Search.aspx |
| 1. Online Atlas of the British and Irish flora \| https://www.brc.ac.uk/plantatlas/ |
| FAO \| http://www.fao.org/geonetwork/srv/en/metadata.show?id=56996 |
| 1. Portal to the Flora of Italy \| 2. http://dryades.units.it/floritaly/index.php?procedure=taxon_page&tipo=all&id=3730 |
| 1. Nadya Wahid \| Personal communication |
| 1. Acta Plantarum - Flora delle regioni italiane \|   https://www.actaplantarum.org/flora/flora_info.php?id=503730 |
| 1. Santiso et al. [^1^](#_ENREF_1) |
| 1. Ribeiro et al. [^2^](#_ENREF_2) |

**Supplementary Table S1.** Data gathering sources.

| **Variable** | **Code** | **Unit** |
| --- | --- | --- |
| Monthly average minimum temperature | tmin | °C * 10 |
| Monthly average maximum temperature | tmax | °C * 10 |
| Annual mean temperature | BIO1 | °C * 10 |
| Mean diurnal range (mean of monthly (max temp - min temp)) | BIO2 | °C * 10 |
| Isothermality (BIO2/BIO7) (* 100) | BIO3 | % |
| Temperature seasonality (standard deviation *100) | BIO4 | - |
| Max. temperature of warmest month | BIO5 | °C * 10 |
| Min. Temperature of Coldest Month | BIO6 | °C * 10 |
| Temperature annual range (BIO5-BIO6) | BIO7 | °C * 10 |
| Mean temperature of wettest quarter | BIO8 | °C * 10 |
| Mean temperature of driest quarter | BIO9 | °C * 10 |
| Mean temperature of warmest quarter | BIO10 | °C * 10 |
| Mean temperature of coldest quarter | BIO11 | °C * 10 |
| Annual precipitation | BIO12 | mm |
| Precipitation of wettest month | BIO13 | mm |
| Precipitation of driest month | BIO14 | mm |
| Precipitation seasonality (coefficient of variation) | BIO15 | % |
| Precipitation of wettest quarter | BIO16 | mm |
| Precipitation of driest quarter | BIO17 | mm |
| Precipitation of warmest quarter | BIO18 | mm |
| Precipitation of coldest quarter | BIO19 | mm |
| Elevation | - | m |
| Slope | - | degrees |

**Supplementary Table S2.** Original set of the 23 environmental variables (climatic and topographic) that were selected for modelling.

| **ID** | **Taxon** | **Country** | **Site name** | **Latitude** | **Longitude** | **Data type** | **Age oldest** | **Period** | **Reference** |
| --- | --- | --- | --- | --- | --- | --- | --- | --- | --- |
| 1 | *Arbutus* | Spain | Navarrés | 39.1000 | -0.6833 | p | 34151 | Late Pleistocene | [^3^](#_ENREF_3) |
| 2 | *A. unedo* | Portugal | Serra do Sicó | 39.9233 | -8.5399 | m | 23920* | Late Pleistocene | [^4^](#_ENREF_4) |
| 3 | *Arbutus* | Spain | Verdeospesoa mire | 43.0550 | -2.8614 | p | 21329 | Late Pleistocene | [^5^](#_ENREF_5) |
| 4 | *Arbutus* | Spain | Siles lake | 38.3890 | -2.5095 | p | 19427 | Late Pleistocene | [^6^](#_ENREF_6) |
| 5 | *A. unedo* | Spain | PRD-4 | 42.5333 | -8.5167 | p | 12451 | Late Pleistocene | [^7^](#_ENREF_7) |
| 6 | *Arbutus* | France | Biot | 43.8000 | 7.1000 | p | 11176 | Early-Middle Holocene | [^8^](#_ENREF_8) |
| 7 | *A. unedo* | Tunisia | Majen Ben H'mida | 37.1333 | 9.0833 | p | 10975 | Early-Middle Holocene | [^9^](#_ENREF_9) |
| 8 | *A. unedo* | Italy | Lago dell'Accesa | 42.9879 | 10.8952 | p | 10388 | Early-Middle Holocene | [^10^](#_ENREF_10) |
| 9 | *Arbutus* | Crete | Aghia Galini | 35.1083 | 24.6944 | p | 10268 | Early-Middle Holocene | [^11^](#_ENREF_11) |
| 10 | *A. unedo* | Spain | Canaleja | 40.9000 | -2.4500 | p | 10241 | Early-Middle Holocene | [^12^](#_ENREF_12) |
| 11 | *Arbutus* | Italy | Lago dell'Accesa | 42.9875 | 10.8924 | p | 10042 | Early-Middle Holocene | [^13^](#_ENREF_13) |
| 12 | *A. unedo* | Corsica | Bastani | 42.0658 | 9.1342 | p | 8971 | Early-Middle Holocene | [^14^](#_ENREF_14) |
| 13 | *Arbutus* | Greece | Lake Voulkaria | 38.8667 | 20.8333 | p | 7969 | Early-Middle Holocene | [^15^](#_ENREF_15) |
| 14 | *Arbutus* | Italy | Pavullo nel Frignano | 44.3201 | 10.8387 | p | 7578 | Early-Middle Holocene | [^16^](#_ENREF_16) |
| 15 | *A. unedo* | France | Embouchac | 43.5664 | 3.9167 | p | 7498 | Early-Middle Holocene | [^17^](#_ENREF_17) |
| 16 | *Arbutus* | Sardinia | Sa Curcurica | 40.4555 | 9.7875 | p | 7219 | Early-Middle Holocene | [^18^](#_ENREF_18) |
| 17 | *Arbutus* | Greece | Lake Lerna | 37.5778 | 22.7281 | p | 7203 | Early-Middle Holocene | [^19^](#_ENREF_19) |
| 18 | *A. unedo* | Portugal | Lagoa Travessa II | 38.3044 | -8.7725 | p | 7122 | Early-Middle Holocene | [^20^](#_ENREF_20) |
| 19 | *Arbutus* | Italy | Lago del Greppo | 44.1199 | 10.6738 | p | 6969 | Early-Middle Holocene | [^21^](#_ENREF_21) |
| 20 | *Arbutus* | Spain | Sierra de Gádor | 36.9316 | -2.9050 | p | 6913 | Early-Middle Holocene | [^22^](#_ENREF_22) |
| 21 | *A. unedo* | France | Saint Sauveur | 43.5664 | 3.9167 | p | 6811 | Early-Middle Holocene | [^23^](#_ENREF_23) |
| 22 | *Arbutus* | Turquia | Ova Gülü | 36.2667 | 29.3000 | p | 6808 | Early-Middle Holocene | [^24^](#_ENREF_24) |
| 23 | *Arbutus* | France | Tourves | 43.4131 | 5.9107 | p | 6783 | Early-Middle Holocene | [^25^](#_ENREF_25) |
| 24 | *A. unedo* | Portugal | Barbaroxa de Baixo | 38.0791 | -8.8098 | p | 6674 | Early-Middle Holocene | [^26^](#_ENREF_26) |
| 25 | *Arbutus* | Israel | Ein Gedi | 31.4189 | 35.3883 | p | 6230 | Early-Middle Holocene | [^27^](#_ENREF_27) |
| 26 | *A. unedo* | Italy | Lago di Massaciuccoli | 43.8378 | 10.3308 | p | 6071 | Early-Middle Holocene | [^10^](#_ENREF_10) |
| 27 | *Arbutus* | Spain | El Sabinar | 38.2000 | -2.1167 | p | 5994 | Early-Middle Holocene | [^28^](#_ENREF_28) |
| 28 | *Arbutus* | Ireland | Derrycunihy Wood | 51.9628 | -9.5819 | p | 5872 | Early-Middle Holocene | [^29^](#_ENREF_29) |
| 29 | *A. unedo* | Italy | Lago di Massaciuccoli | 43.8378 | 10.3308 | p | 5757 | Early-Middle Holocene | [^10^](#_ENREF_10) |
| 30 | *A. unedo* | Tunisia | Majen El Orbi | 37.1492 | 9.0991 | p | 5269 | Early-Middle Holocene | [^9^](#_ENREF_9) |
| 31 | *A. unedo* | Portugal | Lagoa Travessa II | 38.3044 | -8.7725 | p | 5213 | Early-Middle Holocene | [^20^](#_ENREF_20) |
| 32 | *Arbutus* | Greece | Trikhonis 5 | 38.5737 | 21.5454 | p | 5129 | Early-Middle Holocene | [^30^](#_ENREF_30) |
| 33 | *Arbutus* | Spain | Es Grau | 39.9481 | 4.2586 | p | 4720 | Early-Middle Holocene | [^31^](#_ENREF_31) |
| 34 | *Arbutus* | Greece | Litochoro | 40.1389 | 22.5461 | p | 4283 | Early-Middle Holocene | [^32^](#_ENREF_32) |
| 35 | *Arbutus* | Ireland | Rough Island | 52.0313 | -9.5332 | p | 4210 | Early-Middle Holocene | [^33^](#_ENREF_33) |
| 36 | *A. unedo* | Spain | Culazón | 43.2328 | -4.4892 | p | 4160 | Late Holocene | [^34^](#_ENREF_34) |
| 37 | *A. unedo* | Spain | Peña Negra | 40.3347 | -5.7922 | p | 3976 | Late Holocene | [^35^](#_ENREF_35) |
| 38 | *Arbutus* | Croatia | Bokanjacko | 44.1833 | 15.2333 | p | 3964 | Late Holocene | [^36^](#_ENREF_36) |
| 39 | *Arbutus* | Spain | El Maíllo mire | 40.5467 | -6.2097 | p | 3799 | Late Holocene | [^37^](#_ENREF_37) |
| 40 | *Arbutus* | Spain | La Molina mire | 43.3811 | -6.3272 | p | 3675 | Late Holocene | [^38^](#_ENREF_38) |
| 41 | *Arbutus* | Sicilia | Biviere di Gela | 37.0188 | 14.3446 | p | 3437 | Late Holocene | [^39^](#_ENREF_39) |
| 42 | *Arbutus* | Spain | Valdeyernos bog | 39.4411 | -4.0964 | p | 3397 | Late Holocene | [^40^](#_ENREF_40) |
| 43 | *Arbutus* | Ireland | Lough Inchiquin | 51.8072 | -9.6871 | p | 3325 | Late Holocene | [^41^](#_ENREF_41) |
| 44 | *A. unedo* | Spain | Botija bog | 39.6030 | -4.6967 | p | 3127 | Late Holocene | [^42^](#_ENREF_42) |
| 45 | *Arbutus* | Spain | El Payo | 40.2533 | -6.7711 | p | 3100 | Late Holocene | [^43^](#_ENREF_43) |
| 46 | *Arbutus* | Algeria | Bourdim | 36.8033 | 8.2539 | p | 3057 | Late Holocene | [^44^](#_ENREF_44) |
| 47 | *Arbutus* | Ireland | Camillan Wood2 | 52.0184 | -9.5322 | p | 3002 | Late Holocene | [^29^](#_ENREF_29) |
| 48 | *Arbutus* | Sicilia | Gorgo Basso | 37.6000 | 12.6500 | p | 2827 | Late Holocene | [^45^](#_ENREF_45) |
| 49 | *Arbutus* | Tunisia | Djebel El Ghorra | 36.5975 | 8.3947 | p | 2795 | Late Holocene | [^9^](#_ENREF_9) |
| 50 | *A. unedo* | Portugal | Charco da Candieira | 40.3417 | -7.5764 | p | 2555 | Late Holocene | [^46^](#_ENREF_46) |
| 51 | *A. unedo* | Tunisia | Majen El Orbi | 37.1529 | 9.0984 | p | 2501 | Late Holocene | [^9^](#_ENREF_9) |
| 52 | *A. unedo* | Spain | Lanzahíta | 40.2233 | -4.9368 | p | 2434 | Late Holocene | [^47^](#_ENREF_47) |
| 53 | *Arbutus* | Spain | Patateros bog | 39.5972 | -4.6742 | p | 2068 | Late Holocene | [^48^](#_ENREF_48) |
| 54 | *Arbutus* | Spain | Cañada de la Cruz | 38.0675 | -2.6875 | p | 2061 | Late Holocene | [^49^](#_ENREF_49) |
| 55 | *Arbutus* | Crete | Asi Gonia1 | 35.2489 | 24.2782 | p | 1404 | Late Holocene | [^50^](#_ENREF_50) |
| 56 | *Arbutus* | Crete | Asi Gonia2 | 35.2489 | 24.2782 | p | 1242 | Late Holocene | [^50^](#_ENREF_50) |
| 57 | *Arbutus* | Sicilia | Lago di Venere | 36.8169 | 11.9870 | p | 1101 | Late Holocene | [^51^](#_ENREF_51) |
| 58 | *A. unedo* | Ireland | Sheheree Bog | 52.0403 | -9.4809 | p | 849 | Late Holocene | [^52^](#_ENREF_52) |
| 59 | *A. unedo* | Spain | Labradillos mire | 40.3449 | -4.5707 | p | 839 | Late Holocene | [^53^](#_ENREF_53) |
| 60 | *Arbutus* | Greece | Halos I | 39.1606 | 22.8378 | p | 827 | Late Holocene | [^54^](#_ENREF_54) |
| 61 | *Arbutus* | Ireland | Camillan Wood1 | 52.0184 | -9.5322 | p | 403 | Late Holocene | [^33^](#_ENREF_33) |
| 62 | *A. unedo* | Spain | Las Lanchas | 39.5858 | -4.8943 | p | 310 | Late Holocene | [^42^](#_ENREF_42) |
| 63 | *Arbutus* | Spain | Turbera de la Panera Cabras | 40.1658 | -5.7581 | p | 137 | Late Holocene | [^55^](#_ENREF_55) |

**Supplementary Table S3.** Summary of known fossil occurrences of *Arbutus* and *Arbutus unedo* from Pleistocene to present ranked by the age of the oldest record. The pollen data search was made in the Neotoma Paleoecology Database [^56^](#_ENREF_56) by taxon ('Arbutus'=*Arbutus* or 'Arbutus unedo'=*A. unedo*), and only 'pollen' type dataset were found. Ages are indicated according to the chronostratigraphic charts in calibrated years before present (cal BP), except for the macroremains not possible to be calibrated* (number 2). Data type: p = pollen grains, m = macroremains (leaves, fruits, seeds, wood). Age oldest = is the age of the oldest *Arbutus* and *A. unedo* pollen record in the site. According to the pollen record oldest age the following labeling was used: Late Pleistocene (from 34-11.7 ka), Early-Middle Holocene (11.7-4.2 ka), and Late Holocene-Present (4.2-0 ka). Thousand years BP = ka. Coordinates (latitude/longitude) are in WGS84 reference system (EPSG:4326).

**Supplementary References**

1 Santiso, X., Lopez, L., Retuerto, R. & Barreiro Lozano, R. Population structure of a widespread species under balancing selection: the case of *Arbutus unedo* L. *Front Plant Sci* **6**, doi:10.3389/fpls.2015.01264 (2016).

2 Ribeiro, M. M. *et al.* Genetic diversity and divergence at the *Arbutus unedo* L. (Ericaceae) westernmost distribution limit. *PLoS One* **12**, e0175239, doi:10.1371/journal.pone.0175239 (2017).

3 Carrión, J. S. & Van Geel, B. Fine-resolution Upper Weichselian and Holocene palynological record from Navarrés (Valencia, Spain) and a discussion about factors of Mediterranean forest succession. *Rev. Palaeobot. Palyno.* **106**, 209-236, doi:https://doi.org/10.1016/S0034-6667(99)00009-3 (1999).

4 Figueiral, I. & Terral, J. F. Late Quaternary refugia of Mediterranean taxa in the Portuguese Estremadura: charcoal based palaeovegetation and climatic reconstruction. *Quat Sci Rev* **21**, 549-558, doi:<http://dx.doi.org/10.1016/S0277-3791(01)00022-1> (2002).

5 Pérez-Díaz, S. & López-Sáez, J. A. 33. Verdeospesoa mire (Basque Country, Northern Iberian Peninsula, Spain). *Grana* **56**, 315-317, doi:10.1080/00173134.2016.1271824 (2017).

6 Carrión, J. S. Patterns and processes of Late Quaternary environmental change in a montane region of southwestern Europe. *Quat. Sci. Rev.* **21**, 2047-2066, doi:https://doi.org/10.1016/S0277-3791(02)00010-0 (2002).

7 López-Merino, L., Silva Sánchez, N., Kaal, J., López-Sáez, J. A. & Martínez Cortizas, A. Post-disturbance vegetation dynamics during the Late Pleistocene and the Holocene: An example from NW Iberia. *Global and Planetary Change* **92-93**, 58-70, doi:https://doi.org/10.1016/j.gloplacha.2012.04.003 (2012).

8 Nicol-Pichard, S. & Dubar, M. Reconstruction of late-glacial and holocene environments in southeast France based on the study of a 66-m long core from Biot, Alpes Maritimes. *Vegetation History and Archaeobotany* **7**, 11-15, doi:10.1007/bf01393413 (1998).

9 Stambouli-Essassi, S., Roche, E. & Bouzid, S. Evolution de la végétation et du climat dans le nord-ouest de la Tunisie au cours des 40 derniers millénaires. *Geo-Eco-Trop* **31**, 171-214 (2007).

10 Colombaroli, D. *et al.* Response of broadleaved evergreen Mediterranean forest vegetation to fire disturbance during the Holocene: insights from the peri-Adriatic region. *J Biogeogr* **36**, 314-326, doi:https://doi.org/10.1111/j.1365-2699.2008.01987.x (2009).

11 Bottema, S. Palynological investigations on crete. *Rev Palaeobot Palyno* **31**, 193-217, doi:https://doi.org/10.1016/0034-6667(80)90027-5 (1980).

12 Cerrillo-Cuenca, E. & González-Cordero, A. in *From the Origins: The Prehistory of the Inner Tagus Region* (ed Archaeopress) 21-42 (BAR International Series 2219, 2011).

13 Drescher-Schneider, R. *et al.* Vegetation history, climate and human impact over the last 15,000 years at Lago dell’Accesa (Tuscany, Central Italy). *Vegetation History and Archaeobotany* **16**, 279-299, doi:10.1007/s00334-006-0089-z (2007).

14 Lestienne, M. *et al.* Fires and human activities as key factors in the high diversity of Corsican vegetation. *The Holocene* **30**, 244-257, doi:10.1177/0959683619883025 (2019).

15 Jahns, S. The Holocene history of vegetation and settlement at the coastal site of Lake Voulkaria in Acarnania, western Greece. *Vegetation History and Archaeobotany* **14**, 55-66, doi:10.1007/s00334-004-0053-8 (2005).

16 Vescovi, E., Kaltenrieder, P. & Tinner, W. Late-Glacial and Holocene vegetation history of Pavullo nel Frignano (Northern Apennines, Italy). *Rev Palaeobot Palyno* **160**, 32-45, doi:https://doi.org/10.1016/j.revpalbo.2010.01.002 (2010).

17 Puertas, O. Premiers indices polliniques de néolithisation dans la plaine littorale de Montpellier (Hérault, France). *Bulletin de la Société préhistorique française* **96**, 15-20 (1999).

18 Beffa, G. *et al.* Vegetation and fire history of coastal north-eastern Sardinia (Italy) under changing Holocene climates and land use. *Vegetation History and Archaeobotany* **25**, 271-289, doi:10.1007/s00334-015-0548-5 (2016).

19 Jahns, S. On the Holocene vegetation history of the Argive Plain (Peloponnese, southern Greece). *Veg. Hist. Archaeobot.* **2**, 187-203, doi:10.1007/bf00198161 (1993).

20 Mateus, J. E. *Holocene and present-day ecosystems of the Carvalhal Region, Southwest Portugal. PhD thesis* PhD thesis, Universidade de Utreque, (1992).

21 Vescovi, E., Ammann, B., Ravazzi, C. & Tinner, W. A new Late-glacial and Holocene record of vegetation and fire history from Lago del Greppo, northern Apennines, Italy. *Vegetation History and Archaeobotany* **19**, 219-233, doi:10.1007/s00334-010-0243-5 (2010).

22 Carrión, J., Gómez, P., Poveda, J. F., Yll, R. & Chaín-Navarro, C. Holocene vegetation dynamics, fire and grazing in the Sierra de Gádor, southern Spain. *Holocene* **13**, 839-849, doi:10.1191/0959683603hl662rp (2003).

23 Puertas, O. *Evolution de la vegetation depuis le dryas recent dans la plaine littorale de montpellier (herault, france) a partir de l'analyse pollinique. Dynamique naturelle et anthropisation du milieu. PhD thesis* PhD thesis, Université de Franche-Comté, (1997).

24 Bottema, S. & Woldring, H. Late Quaternary vegetation and climate of southwestern Turkey. Part II. *Palaeohistoria 26*, 123-149 (1984).

25 Nicol-Pichard, S. Analyse pollinique d'une séquence tardi et postglaciaire à Tourves (Var, France). *Ecologia mediterranea* **13**, 29-42, doi:https://doi.org/10.3406/ecmed.1987.1609 (1987).

26 Connor, S., Araújo, J., Gomes, S. & Boski, T. *Final Report Project SWIRL. Centro de Investigação Marinha e Ambiental, Universidade do Algarve*. (2013).

27 Litt, T., Ohlwein, C., Neumann, F. H., Hense, A. & Stein, M. Holocene climate variability in the Levant from the Dead Sea pollen record. *Quat Sci Rev* **49**, 95-105, doi:https://doi.org/10.1016/j.quascirev.2012.06.012 (2012).

28 Carrión, J., Yll, E. I., Willis, K. & Gómez, P. Holocene forest history of the eastern plateaux in the Segura Mountains (Murcia, southeastern Spain). *Rev Palaeobot Palyno* **132**, 219-236, doi:https://doi.org/10.1016/j.revpalbo.2004.07.002 (2004).

29 Mitchell, F. J. G. The Vegetational History of the Killarney Oakwoods, SW Ireland: Evidence from Fine Spatial Resolution Pollen Analysis. *J Ecol* **76**, 415-436, doi:10.2307/2260603 (1988).

30 Bottema, S. Palynological investigations in Greece with special reference to pollen as indicator of human activity. *Palaeohistoria 24*, 257–289 (1982).

31 Burjachs, F. *et al.* Overview of environmental changes and human colonization in the Balearic Islands (Western Mediterranean) and their impacts on vegetation composition during the Holocene. *Journal of Archaeological Science: Reports* **12**, 845-859, doi:https://doi.org/10.1016/j.jasrep.2016.09.018 (2017).

32 Athanasiadis, N. Zur postglazialen Vegetationsentwicklung von Litochoro Katerinis und Pertouli Trikalon (Griechenland). *Flora* **164**, 99-132, doi:https://doi.org/10.1016/S0367-2530(17)31791-7 (1975).

33 Mitchell, F. J. G. The Impact of Grazing and Human Disturbance on the Dynamics of Woodland in S. W. Ireland. *Journal of Vegetation Science* **1**, 245-254, doi:10.2307/3235661 (1990).

34 López-Sáez, J. A. *et al.* 20. Culazón, Cantabrian Mountains (northern Spain). *Grana* **52**, 316-318, doi:10.1080/00173134.2013.768700 (2013).

35 Abel-Schaad, D. & López-Sáez, J. A. Vegetation changes in relation to fire history and human activities at the Peña Negra mire (Bejar Range, Iberian Central Mountain System, Spain) during the past 4,000 years. *Vegetation History and Archaeobotany* **22**, 199-214, doi:10.1007/s00334-012-0368-9 (2013).

36 Grüger, E. in *In supplement to: Grüger, E (1996): Vegetational change. Chapman, J; Shiel, R & Batovic, S (eds.): The changing face of Dalmatia. Archaeological and ecological investigations in a Mediterranean Landscape, Leicester University Press, 365 pp, 33-43* (PANGAEA, 1996).

37 Morales-Molino, C., García-Antón, M., Postigo-Mijarra, J. M. & Morla, C. Holocene vegetation, fire and climate interactions on the westernmost fringe of the Mediterranean Basin. *Quat Sci Rev* **59**, 5, doi:10.1016/j.quascirev.2012.10.027 (2013).

38 López-Merino, L., Cortizas, A. M. & López-Sáez, J. A. Human-induced changes on wetlands: a study case from NW Iberia. *Quat Sci Rev* **30**, 2745-2754, doi:https://doi.org/10.1016/j.quascirev.2011.06.004 (2011).

39 Noti, R. *et al.* Mid- and late-Holocene vegetation and fire history at Biviere di Gela, a coastal lake in southern Sicily, Italy. *Vegetation History and Archaeobotany* **18**, 371-387, doi:10.1007/s00334-009-0211-0 (2009).

40 Dorado-Valiño, M., López-Sáez, J. A. & García-Gómez, E. 26. Valdeyernos, Toledo Mountains (central Spain). *Grana* **53**, 315-317, doi:10.1080/00173134.2014.936490 (2014).

41 Mitchell, F. J. G. in *Biogeography of Ireland: past, present, and future* Vol. 2 (eds M J Costello & K S Kelly) 35-44 (Occasional Publication of the Irish Biogeographical Society, 1993).

42 Luelmo Lautenschlaeger, R., López-Sáez, J. A. & Pérez-Díaz, S. 40. Botija, Toledo Mountains (central Spain). *Grana* **57**, 1-3, doi:10.1080/00173134.2017.1400587 (2018).

43 Abel-Schaad, D. *et al.* Evolución de la vegetación en la Sierra de Gata (Cáceres-Salamanca, España) durante el Holoceno reciente. Implicaciones biogeográficas. *Revista Española de Micropaleontología* **41**, 91-105 (2009).

44 Benslama, M. *et al.* Nouvelles contributions à l’histoire tardiglaciaire et holocène de la végétation en Algérie : analyses polliniques de deux profils sédimentaires du complexe humide d’El-Kala. *C R Biologies* **333**, 744-754, doi:https://doi.org/10.1016/j.crvi.2010.08.002 (2010).

45 Tinner, W. *et al.* Holocene environmental and climatic changes at Gorgo Basso, a coastal lake in southern Sicily, Italy. *Quat Sci Rev* **28**, 1498-1510, doi:https://doi.org/10.1016/j.quascirev.2009.02.001 (2009).

46 van der Knaap, W. O. & van Leeuwen, J. F. N. Late Glacial and early Holocene vegetation succession, altitudinal vegetation zonation, and climatic change in the Serra da Estrela, Portugal. *Rev Palaeobot Palyno* **97**, 239-285, doi:https://doi.org/10.1016/S0034-6667(97)00008-0 (1997).

47 López-Sáez, J. A. *et al.* Late Holocene ecological history of Pinus pinaster forests in the Sierra de Gredos of central Spain. *Plant Ecol* **206**, 195, doi:10.1007/s11258-009-9634-z (2010).

48 Dorado-Valiño, M., López-Sáez, J. A. & Gómez, E. Contributions to the European Pollen Database. 21. Patateros, Toledo mountains (central Spain). *Grana* **53**, 171-173 (2014).

49 Carrión, J. S., Munuera, M., Dupré, M. & Andrade, A. Abrupt vegetation changes in the Segura Mountains of southern Spain throughout the Holocene. *J Ecol* **89**, 783-797, doi:https://doi.org/10.1046/j.0022-0477.2001.00601.x (2001).

50 Atherden, M. A. & Hall, J. A. Human impact on vegetation in the White Mountains of Crete since AD 500. *The Holocene* **9**, 183-193, doi:10.1191/095968399673523574 (1999).

51 Calò, C. *et al.* 1200 years of decadal-scale variability of Mediterranean vegetation and climate at Pantelleria Island, Italy. *The Holocene* **23**, 1477-1486, doi:10.1177/0959683613493935 (2013).

52 Mitchell, F. J. G. & Cooney, T. in *Ross Island mining, metal and society in early Ireland* (ed W O'Brien) 481-493 (Bronze Age Studies no. 6, 2004).

53 Robles López, S., Manzano-Rodríguez, S., Pérez-Díaz, S. & López-Sáez, J. A. 35. Labradillos mire, Gregos Range (central Spain). *Grana* **56**, 398-400, doi:10.1080/00173134.2017.1282976 (2017).

54 Bottema, S. in *New Halos: a Hellenistic town in Thessalía, Greece.* (ed H R Reinders) (H&S Publishers, 1988).

55 Abel-Schaad, D., Am, H., L, L. & López-Sáez, J. A. Cabras y quemorros: Tres siglos de cambios en el paisaje de la vertiente extremeña de la Sierra de Gredos. *Revista de estudios extremeños* **65**, 449-478 (2009).

56 Williams, J. W. *et al.* The Neotoma Paleoecology Database, a multiproxy, international, community-curated data resource. *Quaternary Research* **89**, 156-177, doi:10.1017/qua.2017.105 (2018).
